# Prior incarceration and out-of-pocket dental care expenditures among older adults in the United States

**DOI:** 10.1186/s12903-026-08684-x

**Published:** 2026-05-23

**Authors:** Manali Patel, Luis Mijares, Alexander Testa, Dylan B. Jackson, Kyle T. Ganson, Vahed Maroufy, Rafael Samper-Ternent, Ana C. Neumann, Jason M. Nagata

**Affiliations:** 1 Department of Epidemiology, School of Public Health, University of Texas Health Science Center at Houston, Houston, TX United States; 2 Department of Management, Policy, and Community Health, School of Public Health, University of Texas Health Science Center at Houston, Houston, TX United States; 3 Department of Population, Family and Reproductive Health, Johns Hopkins Bloomberg School of Public Health, Baltimore, MD USA; 4 Factor-Inwentash Faculty of Social Work, University of Toronto, Toronto, ON Canada; 5 Department of Biostatistics and Data Science, School of Public Health, University of Texas Health Science Center at Houston, Houston, TX United States; 6 Department of General Practice and Dental Public Health, School of Dentistry, University of Texas Health Science Center at Houston, Houston, TX United States; 7 Division of Adolescent & Young Adult Medicine, University of California, 550 16th Street, #4172, 94158 San Francisco, CA United States

**Keywords:** Oral health, Older adults, Formerly incarcerated, Incarceration, Dental care, Out-of-pocket expenditures, Health and Retirement Study

## Abstract

**Background:**

An accumulating body of research finds that a history of incarceration is associated with poorer oral health and lower dental care utilization. However, no previous research has investigated whether out-of-pocket (OOP) dental care expenditures differ among those with and without a history of incarceration. The purpose of the current study was to examine whether a history of incarceration is associated with OOP dental care expenditures among older adults in the United States.

**Methods:**

We used pooled cross-sectional nationally representative data from the 2012 and 2014 waves of the Health and Retirement Study (HRS), including 11,438 respondents aged 55 and above. A two-part model was employed: a probit model estimated the likelihood of any OOP dental care expenditures among those with and without any lifetime history of incarceration (yes or no), and a generalized linear model (gamma distribution) estimated the relationship between prior incarceration and the amount of expenditure among those with positive costs. Models were adjusted for demographic, socioeconomic, health, and dental-related covariates.

**Results:**

6.8% of the sample reported a history of incarceration. Prior incarceration was not significantly associated with the likelihood of any OOP dental care expenditures or the amount of OOP dental care expenditures. Supplemental analyses found prior incarceration to be associated with a lower likelihood of any OOP dental care expenditures and a lower amount of dental care expenditures among respondents in the lowest quartile of household income.

**Conclusions:**

Despite known disparities in oral health and access, formerly incarcerated older adults do not differ significantly in OOP dental care expenditures compared to never-incarcerated peers on average. Lower OOP dental care expenditures were observed among formerly incarcerated persons at the lowest income quartile. Future research should explore the relationship between prior incarceration and dental care expenditures at other stages of the life course.

## Introduction

Oral health is critical to healthy aging as it affects systemic health and overall quality of life in older adults [1–4]. However, many older adults face a high financial burden and out-of-pocket (OOP) expenditures for dental services [5, 6]. Indeed, among all types of healthcare services, dental care typically involves the greatest financial barriers [5]. Because Medicare does not consider dental care an essential benefit and does not cover routine dental services, many older adults—especially those no longer employed and lacking employer-sponsored dental insurance—experience high OOP dental care expenditures [6, 7].

Notably, a population that exhibits significant oral health disparities and may be uniquely impacted by OOP dental care expenditures is formerly incarcerated older adults. Incarceration has emerged as an important public health issue and contributor to oral health disparities in the United States (US) [8, 9]. With approximately 1.8 million people incarcerated, the US incarcerates more people than any other country [10, 11]. While prisons and jails are still mostly made up of younger adults, the age structure of the incarcerated population has begun to skew older [11–13]; an estimated 1 in 15 older adults living in the general population reports a history of incarceration in their lifetime [14]. Relative to the never-incarcerated population, formerly incarcerated persons exhibit worse oral health, lower dental care utilization, and less stable dental insurance coverage [8, 15–22].

There are reasons to expect that prior incarceration may affect OOP dental care expenditures. First, formerly incarcerated individuals may experience higher OOP dental care expenditures due to elevated oral health needs and limited insurance coverage [9, 17, 19–22]. Second, dental care during incarceration is often inadequate, leading to untreated dental issues that persist after release [9]. Third, formerly incarcerated individuals frequently endure significant economic hardship, including reduced employment opportunities, diminished wages, and lower wealth, which undermines their capacity to secure dental insurance, as well as access and afford care [23–25]. As a result, when formerly incarcerated individuals do seek dental care, they may require more extensive treatment and bear higher OOP costs due to inadequate or absent insurance. Alternatively, incarceration may be associated with lower OOP dental care expenditures due to lower uptake of dental care services among formerly incarcerated older adults [8, 15–20]. If formerly incarcerated individuals delay or avoid dental care altogether due to financial constraints or inability to pay, this can result in lower overall expenditures [26].

Using nationally representative data from the Health and Retirement Study (HRS) on older adults aged 55 and above, the current study is, to the authors’ knowledge, the first to examine the link between prior incarceration and OOP dental care expenditures. The focus on older adults is warranted considering that (a) this group is the fastest growing segment of the incarcerated population [11–13], (b) emerging research documents that older formerly incarcerated adults exhibit worse overall health [27], including oral health [17], and (c) the lack of Medicare coverage for dental procedures means that older adults may be particularly impacted by OOP dental care expenditures [6, 7]. Based on the above-cited literature, the current study is guided by the following two hypotheses:

- H1: Formerly incarcerated older adults will have higher OOP dental care expenditures compared to never-incarcerated individuals.
- H2: Formerly incarcerated older adults will have lower OOP dental care expenditures compared to never-incarcerated individuals.

## Methods

### Data

The data are from the HRS, an ongoing, nationally representative, longitudinal, biennial survey of older adults aged 50 and older in the US. The HRS is conducted by the University of Michigan and is sponsored by the National Institute on Aging. The HRS is conducted every two years, and new participants are enrolled every 6 years through a multistage sampling design to maintain a nationally representative cohort of older adults [28]. In 2012 and 2014, a random subsample of respondents was selected to answer the leave-behind questionnaire (LBQ), which included questions about their incarceration history. Half of the participants were selected to respond to the questionnaire in 2012, and the other half in 2014. The 2012 h LBQ data are representative of those 53 years and older, whereas the 2014 LBQ data represent those 55 years and older [29]. Response rates were 72.7% in the 2012 LBQ and 77.8% in the 2014 LBQ. Accordingly, the sample is restricted to a pooled cross-sectional design composed of respondents aged 55 or older who completed either the 2012 or 2014 LBQ. The University of Texas Health Science Center at Houston Committee for the Protection of Human Subjects deemed this study exempt from human subjects review because the HRS data are publicly accessible and de-identified (Reference #263855). Individuals with missing data were removed from the sample, resulting in a complete-case analysis of 11,438 respondents. Details on the sample selection procedure are provided in Appendix A.

### Dependent variable

OOP dental care expenditures are self-reported spending for dental services over the two years preceding the survey. It is based on the question “About how much did you pay out-of-pocket for dental bills in the last two years?” Responses are provided in US dollars.

### Independent variable

Prior incarceration is based on the following question asked in the LBQ: “Have you ever been an inmate in a jail, prison, juvenile detention center, or other correctional facility?” (1 = Yes, 0 = No) Importantly, this variable captures lifetime prior incarceration that could have occurred at any point in the life course and does not include the time of when an individual entered or was released from incarceration.

### Covariates

Covariates were included to account for potential confounding factors related to prior incarceration and OOP dental care expenditures. These covariates are based on prior research examining prior incarceration and dental care in the HRS [16, 17, 22] and prior studies of OOP dental care expenditures [30]. Sociodemographic characteristics include race/ethnicity (non-Hispanic White, non-Hispanic Black, Hispanic, and other race/ethnicity), biological sex (male/female), and age (55–64, 65–74, 75–84, 85 + years), highest educational attainment (less than high school, GED, high school graduate, some college, college graduate), military veteran (yes or no), and whether a respondent’s mother graduated high school (yes or no). Additionally, measures are included for household income and wealth. Household income is reported for the last calendar year in US dollars and is calculated by summing the respondent’s and spouse’s earnings, pensions and annuities, Social Security Insurance, SSI and Social Security Disability, Social Security retirement, unemployment and workers’ compensation, other government transfers, household capital income, and other income. Wealth is reported in nominal US dollars by calculating the net value of all non-housing assets (e.g., stocks, mutual funds, trusts, checking and savings accounts, bonds and other financial resources) minus total debts. In the current analysis, both income and wealth are reported in quartiles, with higher values indicative of higher income or wealth [14]. Dental-related covariates include whether or not a respondent has dental insurance coverage (yes or no), dental visit within the past two years (yes or no), and edentulism status (yes or no). Health-related covariates include current smoking status (currently smokes v. does not smoke) and a count of the number of self-reported chronic diseases (heart disease, stroke, diabetes, hypertension, arthritis, lung disease).

### Analysis

To examine the association between prior incarceration and OOP dental care expenditures, we employed a two-part modeling strategy, appropriate for healthcare cost data, given its large proportion of zero expenditures and right-skewed distribution of positive values [31]. The model allows for separate estimation of (1) the probability of any OOP dental expenditure, and (2) the amount of expenditure among those with positive costs. The two-part model is preferable to single-equation alternatives such as OLS on log-transformed expenditures or Tobit regression because the zeros in OOP dental expenditures reflect a genuine behavioral decision rather than censored values, and the approach allows variables of interest – including prior incarceration – to exert distinct effects on the participation decision and on the conditional level of spending [31, 32].

The first part uses a probit regression model to estimate the likelihood of reporting any OOP dental care expenditure in the past two years as a function of incarceration history and covariates. In the second part, we fit a generalized linear model (GLM) with a gamma distribution [32] and log link function to model the level of OOP expenditures among those with positive costs. The gamma distribution is selected because it is suitable for analyzing continuous, positive, right-skewed data that represent the structure of the OOP dental care expenditure measure [33, 34]. Analyses were conducted in Stata v. 18 SE using the “twopm” command [35]. First bivariate models were estimated, and then a separate model was estimated adjusting for sociodemographic, socioeconomic, dental-related, and health-related covariates described above. We conducted postestimation using marginal effects to interpret differences in predicted probability of any OOP dental care expenditures and predicted dollar amount of dental care expenditures between formerly incarcerated and never-incarcerated individuals. Following the main analyses, a series of supplemental analyses were conducted employing interaction terms to assess whether the association between prior incarceration and OOP was moderated by edentulism, dental insurance, household income, and wealth.

Given the large sample size and the absence of issues with statistical power, and because the two-part model is not compatible with multiple imputation procedures in Stata, missing data were handled via listwise deletion [36]. Regression analyses accounted for survey weights (*llbwgtr*), clustering (*raehsamp*), and stratification (*raestrat*) using the *svy* command in Stata v. 18 SE.

## Results

Table 1 presents the descriptive characteristics of the sample population (*n* = 11,438). Overall, 57.0% of respondents reported having any OOP dental care expenditures. Among the subsample with OOP dental care expenditures (*n* = 6,308), the average OOP dental care expenditure was $1124.7 (95% CI: 1054.99, 1194.6) over the prior 2 years. The OOP dental care expenditure measure exhibits positive skew (skewness = 7.25, kurtosis = 93.02) [37, 38]. 6.8% of respondents reported a history of incarceration. The sample was predominantly non-Hispanic White (80.7%) and 54.6% female. Nearly half of respondents (46.9%) had dental insurance, and 68.8% had visited a dentist in the past 2 years.

**Table 1:** Descriptive characteristics of the analytic sample, health and retirement study, 2012 And 2014 (Unweighted *N* = 11,438 Weighted *N* = 72,756,131)

Variable	Weighted % / Mean [SD]	Unweighted *n*
Any OOP dental care expenditures		
No	43.0%	5,130
Yes	57.0%	6,308
OOP dental expenditures, USD (*n* = 6,308)	1124.7 [2038.6]	
*Prior incarceration*		
No	93.2%	10,723
Yes	6.8%	715
*Race/ethnicity*		
Non-Hispanic White	80.7%	8,296
Non-Hispanic Black	8.8%	1,667
Hispanic	7.6%	1,174
Non-Hispanic Other	2.9%	301
*Biological sex*		
Female	54.6%	6,789
Male	45.4%	4,649
*Age category*		
55–64	47.3%	4,209
65–74	30.4%	3,613
75–84	16.1%	2,795
85+	6.2%	821
*Educational attainment*		
Less than high school	11.4%	1,583
GED	4.4%	534
High school	27.9%	3,404
Some college	26.5%	2,975
College or above	29.8%	2,942
*Veteran status*		
No	81.6%	9,282
Yes	18.4%	2,156
*Mother’s education*		
Less than high school	43.3%	5,741
High school or higher	56.7%	5,697
*Household income quartile*		
1st	21.7%	2,860
2nd	21.2%	2,860
3rd	25.3%	2,860
4th	31.7%	2,858
*Wealth quartile*		
1st	23.0%	2,861
2nd	24.1%	2,858
3rd	25.3%	2,860
4th	27.7%	2,859
*Dental insurance*		
No	53.1%	6,625
Yes	46.9%	4,813
*Previous dental visit in the past two years*		
No	31.2%	3,920
Yes	68.8%	7,518
*Edentulism*		
No	87.2%	9,755
Yes	12.8%	1,683
*Current smoker*		
No	87.6%	10,138
Yes	12.4%	1,300
*Number of chronic diseases*	1.19 [1.08]	—

*Abbreviations*: *OOP* Out-of-pocket, *SD* Standard deviation

Weighted percentages account for the complex HRS sampling design. OOP dental expenditures reflect self-reported spending over the preceding two years among respondents with any expenditure (n = 6,308). Em dashes (—) indicate values not applicable

Table 2 presents the results from the two-part regression models. The bivariate probit regression results in Model 1 show a significant association between prior incarceration and having any OOP dental care expenditures (β = -0.43, 95% CI: -0.53, -0.33). The unadjusted probit model indicates that 58.1% of never-incarcerated respondents (95% CI = 56.2%, 60.1%) had OOP dental care expenditures compared to 41.2% (95% CI = 37.5%, 44.9%) of previously incarcerated respondents. However, the incarceration variable is not significant in Model 2, the covariate-adjusted probit model (β = 0.01, 95% CI = -0.16, 0.17). The results of the covariate-adjusted probit model indicate that 57.0% (95% CI = 56.2%, 57.8%) of never-incarcerated respondents have OOP dental care expenditures compared to 57.1% (95% CI = 54.6%, 59.6%) among previously incarcerated respondents. Additional stepwise analyses to detect variables contributing to the non-significant association indicate that the results become non-significant after accounting for income and wealth variables. The results of the bivariate gamma regression model (*n* = 6,308) show that prior incarceration is not significantly associated with OOP dental care expenditures (β = -0.02, 95% CI = -0.26, 0.23) or in the covariate-adjusted analysis in Model 4 (β = -0.03, 95% CI = -0.28, 0.23). The results indicate that, in the covariate-adjusted model, those who were never incarcerated have $641.1 (95% CI = 604.2, 678.7) in dental care expenditures, compared to $626.6 (95% CI = 464.4, 788.7) among previously incarcerated respondents.

**Table 2:** Two-part model estimating the association of prior incarceration with out-of-pocket dental care expenditures, health and retirement study, 2012 And 2014

Variable	Probit (*n* = 11,438)		Generalized Linear Model (*n* = 6,308)	
	Model 1: Bivariate	Model 2: Covariate-Adjusted	Model 3: Bivariate	Model 4: Covariate-Adjusted
	β (95% CI)	β (95% CI)	β (95% CI)	β (95% CI)
Prior incarceration	-0.43*** (-0.53,** -0.33)**	0.01 (-0.16,0.17)	-0.02 (-0.26,0.23)	-0.03 (-0.28,0.23)
Sex (ref = female)	—	-0.08 (-0.18,0.02)	—	-0.08 (-0.20,0.04)
*Race/ethnicity* (ref = non-Hispanic White)	—		—	
Non-Hispanic Black	—	-0.38*** (-0.55,**-0.21)**	—	0.07 (-0.16,0.30)
Hispanic	—	-0.03 (-0.18,0.12)	—	0.50*** (0.24,**0.75)**
Non-Hispanic Other	—	-0.20 (-0.42, 0.03)	—	0.32* (0.02,**0.62)**
*Age group* (ref = 55–64)				
65–74	—	0.16* (0.04,**0.28)**	—	0.03 (-0.12,0.18)
75–84	—	0.25** (0.09,**0.40)**	—	0.04 (-0.08,0.16)
85+	—	0.13 (-0.09,0.35)	—	0.03 (-0.23,0.29)
*Education* (ref = less than high school)				
GED	—	0.05 (-0.23,0.32)	—	0.16 (-0.12,0.44)
High school	—	0.26* (0.04,0.47)	—	0.03 (-0.17,0.23)
Some college	—	0.33** (0.09,0.56)	—	0.07 (-0.11,0.26)
College or above	—	0.32** (0.08,0.56)	—	0.15 (-0.04,0.35)
Military Veteran (ref = no)	—	-0.11 (-0.23,0.01)	—	0.05 (-0.10,0.21)
Mother graduated high school (ref = no)	—	0.01 (-0.08,0.09)	—	0.13* (0.03,**0.23)**
*Household income quartile* (ref = 1st )				
2nd	—	0.32*** (0.16,**0.48)**	—	0.12 (-0.02,0.26)
3rd	—	0.29*** (0.14,**0.44)**	—	0.11 (-0.05,0.27)
4th	—	0.23* (0.04,**0.42)**	—	0.18* (0.02,**0.35)**
*Wealth quartile* (ref = 1st )				
2nd	—	0.07 (-0.07,0.21)	—	0.22** (0.07,**0.37)**
3rd	—	0.20** (0.08,**0.33)**	—	0.27*** (0.12,**0.41)**
4th	—	0.12 (-0.03,0.27)	—	0.38*** (0.22,**0.54)**
Dental insurance (ref = no)	—	-0.72*** (-0.83,**-0.62)**	—	-0.29*** (-0.38,** -0.19)**
Dental visit (ref = no)	—	4.49*** (3.87,**5.11)**	—	-2.18*** (-2.39,**-1.96)**
Edentulism (ref = no)	—	-0.36*** (-0.53,**-0.19)**	—	0.51** (0.19,**0.84)**
Current smoker (ref = no)	—	0.08 (-0.09,0.25)	—	0.21* (0.00,**0.42)**
Number of chronic diseases	—	0.03 (-0.02,0.07)	—	0.02 (-0.03, 0.08)

*Abbreviations*: *CI* Confidence interval, *ref* reference category

Probit model estimates the likelihood of any out-of-pocket dental care expenditure in the full sample (*n* = 11,438). Generalized linear model with gamma distribution estimates the OOP dental care expenditure among respondents with any expenditure (*n* = 6,308). Coefficients in bold are statistically significant. * *p* < 0.05; ** *p* < 0.01; *** *p* < 0.001

**Fig. 1:**
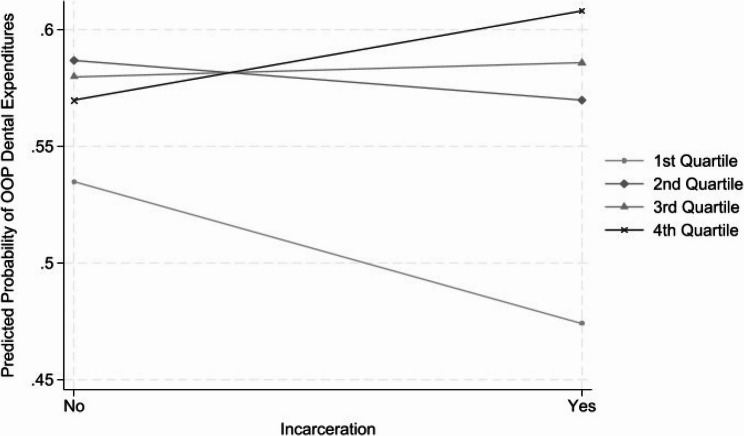
Predicted Out-Of-Pocket Any Dental Care Expenditures of Two-Part Model Regressing Out-Of-Pocket Dental Care Expenditures on Prior Incarceration * Income Quartiles, Health and Retirement Study, 2012 and 2014

**Fig. 2:**
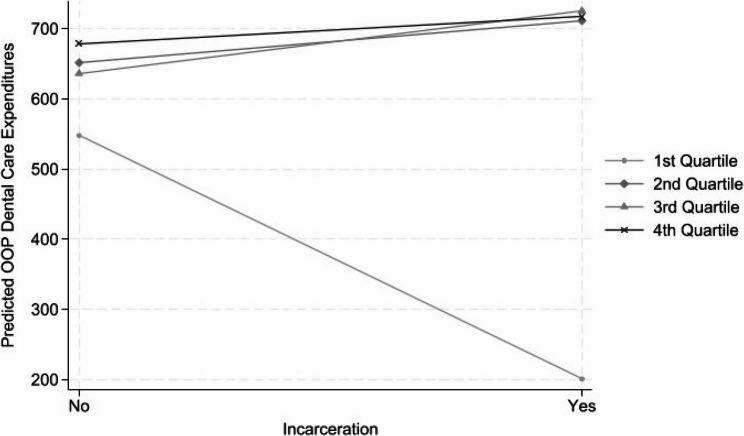
Predicted Out-Of-Pocket Dental Care Expenditures of Two-Part Model Regressing Out-Of-Pocket Dental Care Expenditures on Prior Incarceration * Income Quartiles, Health and Retirement Study, 2012 and 2014

Supplemental analyses were performed to investigate whether the association between prior incarceration and OOP was moderated by key features, including (a) edentulism, (b) dental insurance, (c) household income, and (d) wealth. Across all moderation analyses, the results were consistently null, except for household income (see Appendices B-F). The results from Model 1 show a significant interaction between prior incarceration and income. Expressed as predicted probabilities in Fig. 1 (see also Appendix F), the results indicate the probability of any OOP dental care expenditures is 0.474 (95% CI = 0.401, 0.547) among respondents with prior incarceration who were in the 1st income quartile, compared to 0.535 (95% CI = 0.509, 0.560) of respondents in the 1st income quartile with no prior incarceration history. Next, the results similarly showed a significant interaction between income and incarceration for OOP dental care expenditures. Expressed visually in Fig. 2 (see also Appendix F), the results show that OOP dental care expenditures were similar between those with and without a history of incarceration at all income quartiles – except for the lowest (1st quartile), at which incarceration was associated with significantly lower OOP. Specifically, the model predicts that among respondents at the lowest income quartile, for those without a history of incarceration, OOP dental care expenditures are predicted to be $547.8 (95% CI = 481.0, 614.5), compared to $201.0 (95% CI = 131.4, 270.5) among respondents with a history of incarceration.

## Discussion

This study used national HRS data to assess whether prior incarceration is associated with OOP dental care expenditures among older adults in the US. The results demonstrated no significant differences between prior incarceration and OOP dental care expenditures among the full sample; however, there was an association in moderation analyses examining the interaction between prior incarceration and household income, such that for those at the lowest income quartile, formerly incarcerated older adults had lower odds of any OOP dental care expenditures and lower total expenditures than respondents without a history of incarceration.

The null association between prior incarceration and OOP dental care expenditures can be explained by several possible mechanisms. First, when formerly incarcerated persons use dental care, they may disproportionately receive care in settings that are free or subsidized (e.g., safety-net clinics, federally qualified health centers), effectively reducing their OOP spending levels, despite having greater oral health needs [39]. Second, because OOP dental care expenditures in the HRS reflect only services for which respondents paid, this measure misses care that was recommended but declined due to cost. In other words, formerly incarcerated persons may endure a greater amount of unmet care needs due to an inability to pay for services that are needed [40]. Thus, formerly incarcerated individuals may have more recommended OOP dental care expenditures but do not actually expend more OOP, perhaps because of cost barriers. Third, both formerly incarcerated and never-incarcerated older adults may be similarly constrained in their ability to spend on dental care due to the broader structural insufficiencies in dental coverage for older populations. With traditional Medicare offering limited or no dental benefits, large segments of older adults—regardless of incarceration history—may be limited in their ability to pay for dental care services due to affordability concerns, resulting in similar OOP expenditures across groups [6, 7].

Next, the results of a lower probability of any OOP dental care expenditures and a lower total amount of OOP dental care expenditures among formerly incarcerated older adults in the lowest income quartile can be interpreted through several perspectives. From a cumulative disadvantage perspective [41, 42], formerly incarcerated individuals frequently experience compounding economic and social hardships that constrain their capacity to afford dental care and erode the resources used to pay OOP costs later in life [23–25]. Discrimination and stigma may further shape dental care engagement, as formerly incarcerated individuals can encounter bias in healthcare settings, distrust of providers, or perceived unwelcoming clinical environments that discourage help-seeking and reduce the likelihood of initiating or completing recommended treatment [43–45]. In turn, delayed or foregone care driven by affordability concerns may result in lower OOP dental care expenditures. From a clinical perspective, this pattern may reflect delayed care-seeking. Patients in this group often present when symptoms become severe and may still be unable to proceed with recommended treatment due to cost, resulting in both lower utilization and lower OOP expenditures.

Moving forward, key areas of future research are needed to better understand the relationship between prior incarceration and OOP dental care expenditures. Qualitative research could provide valuable insights into how formerly incarcerated individuals, particularly those of low income, navigate dental care decisions, including how OOP dental care expenditures are factored into decisions about accepting treatment plans and selecting when, where, and whether to receive dental care [46, 47]. Additionally, a promising area of future research would be the use of longitudinal data to examine how OOP dental expenditures vary over time among formerly incarcerated older adults, given evidence of diverse trajectories in dental care use and dental insurance throughout older adulthood [15, 21]. It would also be beneficial to investigate the role of policy changes, such as the Affordable Care Act, in shaping dental insurance coverage and dental reimbursements for OOP dental care expenditures [19, 48]. Of note, however, while prior studies have documented that formerly incarcerated older adults are less likely to have dental insurance [21, 22], there was no significant interaction between incarceration history and dental insurance status on OOP dental care expenditures. It may be that specific features of coverage not captured in our analyses—such as benefit generosity, cost-sharing structures, and payer type—differentially shape OOP dental care expenditures among formerly incarcerated older adults. Future research is needed to further examine how these features of insurance coverage may moderate the relationship between prior incarceration and dental care expenditures.

## Limitations

The current study has limitations, which should be considered when interpreting the results. First, incarceration history was measured through a self-reported measure, which may be subject to recall or social desirability bias [49]. Specifically, in some cases, individuals may answer that they have not been incarcerated due to the social stigma against incarceration [50] or may not have recalled if the incarceration occurred much earlier in life. In addition, this measure does not capture potentially important details such as when in the life course an individual was incarcerated, the type of facility (jail v. prison), the number of incarceration episodes, or how recently an individual was released. Considering the age of the HRS sample, the impacts of incarceration on OOP dental care expenditures may be different in the case that an individual’s incarceration occurred in early adulthood compared to middle or older adulthood.

Second, the measure of OOP dental care expenditures was self-reported over the past two years. Accordingly, this measure may be subject to potential measurement error or recall bias. For instance, individuals may not have recalled the exact amount paid OOP for a dental care procedure, and it is not discernible whether they systematically under- or overestimate this amount. A valuable direction for future work would be to examine the relationship between prior incarceration and OOP dental care expenditures using shorter recall periods (e.g., the past 6 months or 1 year) or dental billing data to obtain a more precise measure of dental care costs. In addition, OOP dental care expenditures were assessed only over a single time point and may not reflect long-term cost patterns. Third, the HRS does not include information on the specific type or purpose of dental visits. As a result, we cannot distinguish whether costs stemmed from preventive care (e.g., cleanings) or more intensive, costly procedures (e.g., extractions, crowns), nor whether OOP dental care expenditures stemming from different types of dental services vary by incarceration history.

Fourth, although dental insurance status was included as a covariate, the HRS lacks detail on the nature of coverage (i.e., Medicaid, a private plan, or Medicare Advantage), which may influence cost-sharing arrangements and OOP dental care expenditures. Fifth, due to the HRS sampling frame of older adults, the results may be impacted by survivorship bias, whereby older, formerly incarcerated adults may be a healthier group on average. This may be impactful in the case of incarceration, as prior studies find that formerly incarcerated individuals have an average age of death of less than 50 years old [51, 52]. Additionally, OOP dental care expenditures reflect only realized spending and do not capture unmet or deferred care, which may be particularly relevant among financially vulnerable populations. Finally, we could not examine contextual influences, such as neighborhood-level socioeconomic disadvantage, geographic availability of dental providers, or differences in local dental safety-net infrastructure. Future research would benefit from longitudinal, multilevel data that capture individual, social, and geographic influences over time to develop a more comprehensive picture of dental care expenditures over the life course.

This study also has several strengths. To the authors’ knowledge, it is the first to examine the association between prior incarceration and OOP dental care expenditures among older adults. The use of the HRS, a large, nationally representative survey of older adults in the US, enhances the generalizability of findings. In addition, the analyses adjusted for a broad range of sociodemographic, socioeconomic, health, and dental-related covariates, and supplemental analyses examined effect modification by income, wealth, dental insurance, and edentulism, providing a more nuanced understanding of how the relationship between prior incarceration and OOP dental expenditures may vary across subgroups.

## Conclusion

The current study aimed to extend prior research on the relationship between prior incarceration and dental care-related outcomes by examining the association between prior incarceration and OOP dental care expenditures. While there was no association between prior incarceration and OOP dental care expenditures in the full sample, the results indicated that formerly incarcerated individuals in the lowest income quartile had a lower probability of any OOP dental care expenditures and less OOP dental care expenditures than their never-incarcerated counterparts. As the first study to examine this relationship, future research is needed to disentangle how incarceration history influences dental care expenditures. Studies incorporating clinical, administrative, and contextual data, as well as mixed-methods studies, are critical avenues for advancing research on how incarceration influences facets of dental care use.

## Data Availability

The datasets generated and/or analyzed during the current study are publicly available. Data used in this study can be obtained at: https://hrsdata.isr.umich.edu/data-products. Data and code will be made publicly available following publication at ICPSR: https://www.openicpsr.org/openicpsr/project/214263/version/V3/view. Queries about the data can be directed to Alexander Testa: [alexander.testa@uth.tmc.edu]

## Appendix A

**Table 3:** Sample selection procedure

Criteria	Sample Size
Participated in the 2012 or 2014 LBQ	*n* = 19,628
	↓
Had a Response on Prior Incarceration	*n* = 14,460
	↓
Had a response on Dental OOP	*n* = 14,452
	↓
Had Response on Covariates	*n* = 11,611
	↓
Had Valid Survey weights	*n =* 11,438

*LBQ* Leave-Behind Questionnaire

## Appendix B

**Table 4:** Two-part model of the association between prior incarceration and out-of-pocket dental care expenditures, with edentulism as a moderator, health and retirement study, 2012 and 2014

Variable	Model 1: Probit (*n* = 11,438)	Model 2: Generalized Linear Model (*n* = 6,308)
	β (95% CI)	β (95% CI)
Prior incarceration	0.07 (-0.12, 0.26)	-0.02 (-0.28, 0.24)
Edentulism	**-0.30**(-0.48**,** -0.11)**	**0.52** (0.18**,** 0.86)**
Prior incarceration × Edentulism	-0.70 (-1.41, 0.01)	-0.11 (-0.66, 0.43)
Covariates	*Included*	*Included*

Model 1 is a probit model estimating the likelihood of any out-of-pocket (OOP) dental care expenditure in the full sample (*n* = 11,438). Model 2 is a generalized linear model with gamma distribution and log link estimating the level of OOP expenditure among respondents with any expenditure (*n* = 6,308). Both models adjust for race/ethnicity, biological sex, age group, educational attainment, military veteran status, mother’s education, household income quartile, wealth quartile, dental insurance, dental visit in the past two years, edentulism, current smoking status, and number of chronic diseases (covariate estimates suppressed). Coefficients in bold are statistically significant. * *p* < 0.05; ** *p* < 0.01; *** *p* < 0.001

## Appendix C

**Table 5:** Two-part model of the association between prior incarceration and out-of-pocket dental care expenditures, with dental insurance as a moderator, health and retirement study, 2012 And 2014

Variable	Model 1: Probit (*n* = 11,438)	Model 2: Generalized Linear Model (*n* = 6,308)
	β (95% CI)	β (95% CI)
Prior incarceration	0.15 (-0.12, 0.43)	-0.03 (-0.29,0.23)
Dental insurance	**-0.71*** (-0.82**,** -0.60)**	**-0.29*** (-0.39**,** -0.19)**
Prior incarceration × Dental insurance	-0.23 (-0.62, 0.17)	0.01 (-0.52, 0.54)
Covariates	*Included*	*Included*

Model 1 is a probit model estimating the likelihood of any out-of-pocket (OOP) dental care expenditure in the full sample (*n* = 11,438). Model 2 is a generalized linear model with gamma distribution and log link estimating the level of OOP expenditure among respondents with any expenditure (*n* = 6,308). Both models adjust for race/ethnicity, biological sex, age group, educational attainment, military veteran status, mother’s education, household income quartile, wealth quartile, dental insurance, dental visit in the past two years, edentulism, current smoking status, and number of chronic diseases (covariate estimates suppressed). Coefficients in bold are statistically significant. * *p* < 0.05; ** *p* < 0.01; *** *p* < 0.001

## Appendix D

**Table 6:** Two-part model of the association between prior incarceration and out-of-pocket dental care expenditures, with household income quartile as a moderator, health and retirement study, 2012 And 2014

Variable	Model 1: Probit (*n* = 11,438)	Model 2: Generalized Linear Model (*n* = 6,308)
	β (95% CI)	β (95% CI)
Prior incarceration	-0.29 (-0.61, 0.03)	**-0.89*** (-1.21**,** -0.57)**
*Household income quartile*		
1st (ref)	—	—
2nd	**0.31*** (0.14**,** 0.47)**	0.08 (-0.06, 0.22)
3rd	**0.26*** (0.11**,** 0.41)**	0.07 (-0.09, 0.23)
4th	**0.20* (0.01**,** 0.39)**	0.15 (-0.02, 0.33)
*Prior incarceration × Income quartile*		
Prior incarceration × 1st (ref)	—	—
Prior incarceration × 2nd	0.18 (-0.34, 0.71)	**1.01*** (0.50**,** 1.51)**
Prior incarceration × 3rd	0.33 (-0.18, 0.84)	**1.01*** (0.49**,** 1.53)**
Prior incarceration × 4th	**0.56* (0.05**,** 1.07)**	**0.88*** (0.26**,** 1.49)**
Covariates	*Included*	*Included*

Model 1 is a probit model estimating the likelihood of any out-of-pocket (OOP) dental care expenditure in the full sample (*n* = 11,438). Model 2 is a generalized linear model with gamma distribution and log link estimating the level of OOP expenditure among respondents with any expenditure (*n* = 6,308). Both models adjust for race/ethnicity, biological sex, age group, educational attainment, military veteran status, mother’s education, household income quartile, wealth quartile, dental insurance, dental visit in the past two years, edentulism, current smoking status, and number of chronic diseases (covariate estimates suppressed). Coefficients in bold are statistically significant. * *p* < 0.05; ** *p* < 0.01; *** *p* < 0.001

## Appendix E

**Table 7:** Two-part model of the association between prior incarceration and out-of-pocket dental care expenditures, with wealth quartile as a moderator, health and retirement study, 2012 And 2014

Variable	Model 1: Probit (*n* = 11,438)	Model 2: Generalized Linear Model (*n* = 6,308)
	β (95% CI)	β (95% CI)
Prior incarceration	-0.08 (-0.41, 0.25)	-0.07 (-0.48, 0.35)
*Wealth quartile*		
1st (ref)	—	—
2nd	0.06 (-0.07, 0.20)	**0.22** (0.05**,**0.38)**
3rd	**0.19* (0.06**,**0.32)**	**0.26** (0.10**,** 0.41)**
4th	0.11 (-0.04, 0.26)	**0.37*** (0.22**,** 0.53)**
*Prior incarceration × Wealth quartile*		
Prior incarceration × 1st (ref)	—	—
Prior incarceration × 2nd	0.04 (-0.48, 0.55)	0.01 (-0.73, 0.74)
Prior incarceration × 3rd	0.19 (-0.34, 0.71)	0.13 (-0.34, 0.60)
Prior incarceration × 4th	0.17 (-0.45, 0.80)	0.03 (-0.88, 0.95)
Covariates	*Included*	*Included*

Model 1 is a probit model estimating the likelihood of any out-of-pocket (OOP) dental care expenditure in the full sample (*n* = 11,438). Model 2 is a generalized linear model with gamma distribution and log link estimating the level of OOP expenditure among respondents with any expenditure (*n* = 6,308). Both models adjust for race/ethnicity, biological sex, age group, educational attainment, military veteran status, mother’s education, household income quartile, wealth quartile, dental insurance, dental visit in the past two years, edentulism, current smoking status, and number of chronic diseases (covariate estimates suppressed). Coefficients in bold are statistically significant. * *p* < 0.05; ** *p* < 0.01; *** *p* < 0.001

## Appendix F

**Table 8:** Predicted margins of out-of-pocket dental care expenditures and amount of out-of-pocket dental care expenditures by income quartile and prior incarceration status, health and retirement study, 2012 and 2014

Income Quartile	No Prior Incarceration	Prior Incarceration
Predicted Margins of Any OOP Dental Care Expenditures		
1st	0.535 (0.509, 0.560)	0.474 (0.401, 0.547)
2nd	0.587 (0.571, 0.602)	0.569 (0.504, 0.636)
3rd	0.580 (0.567, 0.593)	0.586 (0.535, 0.636)
4th	0.569 (0.553, 0.586)	0.608 (0.561, 0.655)
Predicted Margins of OOP Dental Care Expenditures		
1st	$547.8 (481.0, 614.5)	$201.0 (131.4, 270.5)
2nd	$651.5 (574.0, 728.9)	$711.4 (368.5, 1054.4)
3rd	$636.0 (569.1, 702.8)	$725.1 (410.3, 1040.0)
4th	$678.6 (602.3, 754.8)	$716.7 (365.1, 1068.3)

Results show the predicted value from Appendix D and Figs. 1 and 2. 95% confidence intervals are in parentheses
